# Predictive Power of Oxygen Desaturation Index (ODI) and Apnea-Hypopnea Index (AHI) in Detecting Long-Term Neurocognitive and Psychosocial Outcomes of Sleep-Disordered Breathing in Children: A Questionnaire-Based Study

**DOI:** 10.3390/jcm12093060

**Published:** 2023-04-23

**Authors:** Marco Zaffanello, Giuliana Ferrante, Leonardo Zoccante, Marco Luigi Ciceri, Luana Nosetti, Laura Tenero, Michele Piazza, Giorgio Piacentini

**Affiliations:** 1 Department of Surgical Sciences, Dentistry, Gynecology and Pediatrics, University of Verona, 37126 Verona, Italy; giuliana.ferrante@univr.it (G.F.); laura.tenero@aovr.veneto.it (L.T.); michele.piazza@univr.it (M.P.); giorgio.piacentini@univr.it (G.P.); 2 Child and Adolescent Neuropsychiatry Unit, Maternal-Child Integrated Care Department, Integrated University Hospital Verona, 37126 Verona, Italy; leonardo.zoccante@aovr.veneto.it (L.Z.); marco.ciceri@aulss9.veneto.it (M.L.C.); 3 Department of Pediatrics, Pediatric Sleep Disorders Center, F. Del Ponte Hospital, Insubria University, 21100 Varese, Italy; luana.nosetti@uninsubria.it

**Keywords:** children, home respiratory polygraphy, obstructive apnea–hypopnea index, oxygen desaturation index, quality of life, questionnaires, sleep disordered breathing

## Abstract

Pediatric obstructive sleep apnea can negatively affect children’s neurocognitive function and development, hindering academic and adaptive goals. Questionnaires are suitable for assessing neuropsychological symptoms in children with sleep-disordered breathing. The study aimed to evaluate the effectiveness of using the Oxygen Desaturation Index compared to the Obstructive Apnea–Hypopnea Index in predicting long-term consequences of sleep-disordered breathing in children. We conducted a retrospective analysis of respiratory polysomnography recordings from preschool and school-age children (mean age: 5.8 ± 2.8 years) and followed them up after an average of 3.1 ± 0.8 years from the home-based polysomnography. We administered three validated questionnaires to the parents/caregivers of the children by phone. Our results showed that children with an Oxygen Desaturation Index (ODI) greater than one event per hour exhibited symptoms in four domains (physical, school-related, Quality of Life [QoL], and attention deficit hyperactivity disorder [ADHD]) at follow-up, compared to only two symptoms (physical and school-related) found in children with an Obstructive Apnea–Hypopnea Index greater than one event per hour at the time of diagnosis. Our study also found a significant correlation between the minimum SpO_2_ (%) recorded at diagnosis and several outcomes, including Pediatric Sleep Questionnaire (PSQ) scores, physical, social, and school-related outcomes, and ADHD index at follow-up. These results suggest that the Oxygen Desaturation Index could serve as a valuable predictor of long-term symptoms in children with sleep-disordered breathing, which could inform treatment decisions. Additionally, measuring minimum SpO_2_ levels may help assess the risk of developing long-term symptoms and monitor treatment outcomes.

## 1. Introduction

It has been estimated that between 1% and 5% of children suffer from sleep-disordered breathing (SDB) [1]. This problem can cause various disorders, including growth deficits, neurocognitive and behavioral abnormalities, and pulmonary and heart disease [2,3]. In particular, pediatric obstructive sleep apnea (OSA) from moderate to severe can harm neurocognitive function and hinder children’s development, affecting their ability to achieve academic and adaptive skills, altering their learning abilities, and delaying their independence [4]. Several studies have demonstrated a clear association between OSA and behavioral disorders such as impulse control disorder, inattention, and oppositional defiant disorder [5]. Some of these studies have also found a correlation between the severity of OSA and the degree of neuropsychological deficits in children [6,7]. Research has shown that improving SDB screening and adopting effective management strategies can positively impact young patients’ long-term health and development [1].

Questionnaires are suitable tools for assessing neuropsychological symptoms in children with SDB [8,9]. These instruments are often used as the first step in symptom evaluation, as they provide a preliminary assessment of symptom severity and etiology. However, questionnaires alone are not sufficient for a definitive diagnosis of SDB. Typically, they are used in conjunction with other assessment tools, such as polysomnography (PSG), which provides more precise information on the severity of SDB [10]. However, some questionnaires may be helpful as an initial screening when PSG is not available or feasible [7,11].

The overnight PSG is considered the gold standard for diagnosing SDB [12,13]. In PSG, the main parameter used to diagnose SDB is the AHI [14]. Some studies have examined the usefulness of polysomnographic parameters in predicting long-term complications of OSA. For example, the presence of SDB in children has been associated with growth delay [15,16]. Additionally, an increase in obstructive AHI (oAHI) in infants has been linked to lower behavioral scores at the age of three years [17]. It has also been demonstrated that children with severe SDB are more likely to experience persistent OSA and its complications [18,19].

In addition, blood oxygenation levels, measured by minimum and mean peripheral capillary oxygen saturation (SpO_2_), may increase the risk of hypertension in adults [20]. The oxygen desaturation index (ODI) may be a good indicator of the presence and severity of OSA in children [21]. In particular, the ODI could be a useful parameter for evaluating the correct oAHI in patients with severe OSA [22]. A review article has reported the results of PSG studies in children aged between 5 and 12.9 years. The article suggests that questionnaires on sleep-related quality of life (QoL), such as PSQ-SRBD and OSA-18, may show improvement after adenotonsillectomy if positive changes occur in sleep parameters, such as AHI, ODI, and SpO_2_, during follow-up (3 to 13 months) in children with OSAS [23].

Intermittent desaturations during sleep have multi-organ implications secondary to oxidative damage from free oxygen radicals [24]. The reductions in SpO_2_ that occur during sleep can interfere with the normal maturation of the central nervous system, causing cognitive and behavioral problems [25]. However, currently, there are no studies that have evaluated whether ODI is a good assessment parameter for long-term neurobehavioral complications in children.

The current study aims to evaluate the effectiveness of using the ODI compared to the oAHI in predicting the long-term neurocognitive and psychosocial consequences of SDB in children.

## 2. Materials and Methods

This is a retrospective and cross-sectional study that enrolled a group of preschool and school-age children with varying OSA severity. Enrollment was defined based on the availability of data that were collected retrospectively, as well as the voluntary willingness of patients to participate. The study included Caucasian children of both genders who underwent cardiorespiratory PSG between 2015 and 2019 at the Pediatrics Department of the University of Verona, Italy. Children with incomplete or missing home recordings, recordings lasting less than 6 h, and/or comorbidities such as neurological or neuromuscular diseases, syndromic genetic disorders, and neuropsychiatric syndromes were excluded from the study. The Ethics Committee approved the protocol for clinical trials in the provinces of Verona and Rovigo at Integrated University Hospital (CESC601). All parents/caregivers gave informed consent for the scientific use of the data.

### 2.1. Study Population

**Figure 1:**
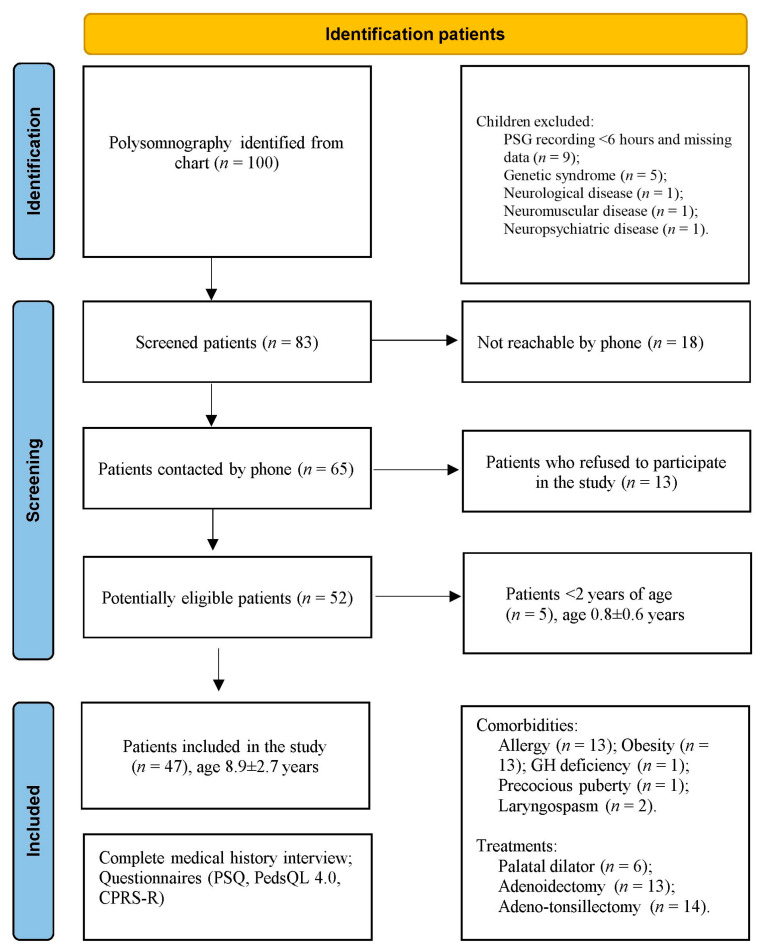
Children enrolled, included, and excluded in the study and their characteristics.

One hundred children who had undergone home PSG during the night between 2015 and 2019 were recruited (Figure 1). Initially, nine children with a registration duration of less than 6 h or missing data were excluded from the database, and five children were excluded from the statistics because they were less than two years old (0.8 ± 0.6, range 0.4–1.9 years) at enrolment (T_0_). Furthermore, eight children were excluded due to genetic (*n* = 5), neurological (*n* = 1), neuromuscular (*n* = 1), and neuropsychiatric (*n* = 1) conditions. Therefore, 83 patients (T_0_; 59% males) were contacted. At follow-up (T_1_), 18 families did not respond to phone calls, and 13 responded but refused to participate in the study. Fifty-two patients (63.5% males) agreed to participate in the survey by answering all three questionnaires.

### 2.2. Anthropometry

Height and weight were measured using a precision medical scale (WUNDER C201, Wunder Sa.Bi. srl, Milan, Italy) and a telescopic stadiometer. Specially trained healthcare personnel measured the children while wearing light clothing and no shoes. Weight was recorded with an accuracy of 0.1 kg, and height was measured with an accuracy of 0.1 cm.

Body mass index (BMI) was calculated as weight (kg) divided by height squared (m^2^). BMI percentiles, and BMI z-scores were calculated using an online tool (http://www.bcm.edu/bodycomplab/BMIapp/BMI-calculator-kids.html; accessed on 1 September 2022), which is based on CDC growth charts for children and adolescents aged 2 to 19 years (https://www.cdc.gov/healthyweight/bmi/calculator.html; accessed on 1 September 2022).

### 2.3. Respiratory Polysomnography

The individual physiological Home Respiratory Polysomnography (PSG) recordings were stored in a designated database, which includes the analysis software.

Overnight PSG recordings were conducted using a portable ambulatory device (SOMNOscreenTM PSG, SOMNOmedics GmbH, Randersacker, Germany) with continuous monitoring of various physiological parameters, including nasal airflow via cannula, chest and abdominal respiratory movements using belts, oxygen saturation (SpO_2_) via digital pulse oximetry, heart rate and electrocardiogram (ECG), body position using a mercury sensor, and tracheal sounds using a microphone, as previously described [26]. The device was programmed to turn on and off automatically according to the child’s sleep habits. 

Parents were trained to use the device properly, and they recorded in a diary the time their child fell asleep, nocturnal awakenings, and the time of morning awakening.

The recordings were initially analyzed by the DOMINO software (Somnomedics v.2.6.0, Randersacker, Germany) and then carefully reviewed by one of the investigators (MZ). The estimated total sleep time (eTST) was calculated according to published criteria [14,27] and served as the denominator for any index calculation. Respiratory events were evaluated according to the American Academy of Sleep Medicine guidelines [14]. The oAHI was defined as the sum of obstructive apnea (OA), mixed apneas, and hypopneas divided by eTST. An oAHI ≤ 1 event/h. of eTST was defined as normal, and oAHI > 1 event/h. of eTST was defined as abnormal [28]. The oxygen desaturation index ≤ 3% (ODI3%) was calculated as the total number of desaturations divided by eTST (event/h.). The mean and minimum SpO_2_ (%) were also automatically calculated. Snoring events (expressed as a percentage of eTST) were also calculated in the overall nocturnal recording [14]. 

### 2.4. Telephone Interview

The parents/caregivers of the children were contacted by phone between February and June 2021, during the third wave of the COVID-19 pandemic. Those who voluntarily agreed to participate provided their medical history, including the presence of comorbidities, allergies, and whether they had undergone adenoid or tonsil surgery in the years following the examination. Three validated questionnaires were then administered by phone: the Pediatric Sleep Questionnaire in its shortened 22-item version (PSQ-SRBD), the Pediatric Quality of Life Inventory (PedsQL 4.0), and the abbreviated version of the Con-ners’ Parent Rating Scale-Revised (CPRS-R). Time 0 (T_0_) was defined as the moment of the overnight PSG recording, while time 1 (T_1_) was when the patients were contacted for follow-up by phone about three years later. 

### 2.5. Questionnaires

At follow-up, the parents of the children completed the PSQ, PedsQL, and CRS-R questionnaires at home.

### 2.6. Pediatric Sleep Questionnaire

The PSQ is utilized to screen obstructive sleep apnea syndrome (OSAS) and assess a patient’s quality of life (QoL) [29,30]. It consists of 22 questions that require a “yes”, “no”, or “don’t know” response. The overall score is computed by determining the percentage of affirmative responses (“yes”), and a result is deemed significant if the number of positive responses exceeds 33% of the total. 

### 2.7. Pediatric Quality of Life Inventory

PedsQL is a questionnaire for assessing QoL in children and adolescents between the ages of 2 and 18 years [31,32]. The questionnaire is suitable for a wide range of ages and is validated in the Italian language [32]. It is adapted to the patient’s age and divided into patients aged 2–4 years, 5–7 years, 8–12 years, and 13–18 years. It consists of 23 questions divided into 4 domains: health and physical activity (8 questions), emotions (5 questions), social relationships (5 questions), and school-related (5 questions). The first eight questions are summarized as a physical health score, while the remaining 15 fall under the psychosocial health assessment. To answer the questions, parents must refer to their child’s life in the month preceding the administration of the questionnaire [33,34]. The highest score corresponds to the higher QoL. 

### 2.8. Conners’ Parent Rating Scales Revised

The CPRS-R was designed in 1998 to evaluate conditions such as ADHD and ADHD-like disorders. The questionnaire investigates behavioral and cognitive alterations, including hyperactivity, violent behavior, impulsivity, oppositionality, difficulties in behavioral control, attention deficits, and working memory alterations of children and adolescents aged between 3 and 17 years [35]. Parents who fill out the questionnaire should refer to their child’s behavior in the last month. Four possible answers can be given to each question. A score below 60 is considered not indicative of pathology, suggestive between 60 and 70, and indicative of pathology above 70 points [7]. Subjects who obtain a high value in opposition are more likely to violate rules, have problems with authority figures, and easily become annoyed. Patients with slower learning, organizational problems, difficulty completing tasks, and concentration problems score higher in the group investigating cognitive problems. The third index is hyperactivity, manifested in subjects who have difficulty sitting still or doing the same task for a long time and are more restless and impulsive. The ADHD index identifies children and adolescents at risk for ADHD and may be helpful as a screening tool. The raw scores obtained are finally standardized by calculating the T variable, allowing comparison with the reference values and the general population. The profile sheet used to assign scores and for final conversion into standardized T scores takes gender diversity into account. Higher T scores represent worse behavior in each specific category [35].

### 2.9. Statistics

Descriptive and non-parametric (Mann–Whitney U test) analyses were used as statistical methods. The non-parametric Mann–Whitney test was used for two values, providing means and standard deviations (SD) for different outcome measures. Additionally, minimum and maximum values were reported for each variable when appropriate. *p*-values were reported for each outcome measure. If the *p*-value is less than 0.05, the difference between the means is considered statistically significant.

Multiple regression analysis was used to explore the relationship between independent variables (SDB indices) and dependent variables. The beta coefficients, t-values, and significance levels are used to evaluate strength, direction, and statistical significance of the relationship between variable (*p* < 0.05).

The data were recorded into a Microsoft^®^ Excel^®^ database for Windows 11 and statistically analyzed using SPSS version 22.0 for Windows (SPSS Inc., Chicago, IL, USA).

## 3. Results

Table 1 displays descriptive statistics for a sample of patients who were contacted or included in the study. Patients included in the study performed the respiratory PSG at a younger age and had lower height and weight compared to the excluded patients. There were no significant differences in respiratory PSG between included and excluded patients, and their snoring levels were similar.

### Follow-Up

The children were followed up at 8.9 ± 2.7 years after 3.1 ± 0.8 years from the home-based PSG (T_0_). Allergy was reported in 13 patients (27.7%). A total of 36% of the patients had the following comorbidities: obesity (*n* = 13), Growth Hormone deficiency (*n* = 1), precocious puberty (*n* = 1), and laryngospasm (*n* = 2). Six patients (12.8%) used a palatal dilator. A total of 57.5% of patients underwent adenoid and/or tonsil surgery between diagnosis (T_0_) and interview (T_1_): adenoidectomy (*n* = 13), tonsillectomy (*n* = 1), adenotonsillectomy (*n* = 13).

The PSQ showed a total mean score of 35.9 ± 19.6 (range 0–77.3) %. A total of 44.7% of children had a pathological score (>33% of questions positive). The PedsQL 4.0 showed a score of 88 ± 13 (range 46.9–100) % in physical domain, 67.7 ± 16 (range 30–100) % in the emotional domain, 83.8 ± 18.3 (range 35–100) % in the social domain, and 80.4 ± 19.4 (range 25–100) % in the academic domain. The psychosocial health score was 77.3 ± 14.7 (range 30–96.7) %, and the overall QoL score was 81 ± 13.1 (range 47.8–95.7) %.

The CRS-R showed an oppositionality T score of 61.6 ± 14.4 (range 38–98), a cognitive disorder T score of 57.1 ± 15.5 (range 41–95), a hyperactivity T score of 59.6 ± 14.0 (range 39–84), and ADHD index T score of 64.2 ± 14.9 (range 38–94).

Table 2 shows the results of questionnaires administrated to children with oAHI > 1 event/h. on respiratory PSG at diagnosis (T_0_). Children with a positive PSQ score had worse physical results (82.4 ± 13.9%) than those with a negative PSQ score (92.7 ± 10.7%; *p* = 0.015). School performance was also worse in children with a positive PSQ score (74.8 ± 21.1%) compared to those with a negative PSQ score (90.9 ± 8.8%; *p* = 0.048). The table also shows the results of questionnaires administrated to children with ODI > 1 event/h. on respiratory PSG at diagnosis (T_0_). Physical results were worse in children with a positive PSQ score (83.2 ± 14.4%) compared to those with a negative PSQ score (92.9 ± 11.0%; *p* = 0.034). School performance was worse in children with a positive PSQ score (71.2 ± 20.5%) compared to those with a negative PSQ score (90.5 ± 8.8%; *p* = 0.009). Moreover, the ADHD index was worse in children with a positive PSQ score (70.7 ± 12.4%) compared to those with a negative PSQ score (58.7 ± 11.8%; *p* = 0.011).

Table 3 shows the results of questionnaires administrated to children with oAHI > 1 event/h. on respiratory polygraphy at diagnosis (T_0_) who underwent surgery before neuropsychological follow-up. Physical results were worse in children with a positive PSQ score (82.6 ± 16.0%) than those with a negative PSQ score (97.2 ± 4.3%; *p* = 0.007). The total QoL score was worse in children with a positive PSQ score (75.4 ± 14.3%) than those with a negative PSQ score (87.3 ± 7.9%; *p* = 0.030).

The table also shows the results of questionnaires administrated to children with ODI > 1 event/h. on respiratory PSG at diagnosis (T_0_) who underwent surgery before neuropsychological follow-up. Physical results were worse in children with a positive PSQ score (83.1 ± 16.5%) than those with a negative PSQ score (98.4 ± 2.9%; *p* = 0.012). School performance was worse in children with a positive PSQ score (72.5 ± 19.7%) than those with a negative PSQ score (91.3 ± 8.8%; *p* = 0.039). The total QoL score was worse in children with a positive PSQ score (75.9 ± 14.9%) than those with a negative PSQ score (90.1 ± 4.3%; *p* = 0.020). Moreover, the ADHD index was worse in children with a positive PSQ score (71.5 ± 14.7%) than those with a negative PSQ score (55.9 ± 7.1%; *p* = 0.020).

Table 4 shows a significant linear regression analysis between the PSQ score (%) and physical, emotional, school, and opposition domains (%) at follow-up. Specifically, SDB was associated with decreased physical and emotional results, as well as decreased school results, and increased oppositional behavior. Moreover, a significant negative linear regression was observed between oAHI at diagnosis and physical results at follow-up. Furthermore, the minimum SpO_2_ (%) at diagnosis was significantly correlated with the PSQ scores (%), social and school results (with a positive trend), and ADHD index (with a positive trend) at follow-up but was negatively correlated with physical abilities. In summary, the severity of SDB (measured with SpO_2_ min and oAHI) at diagnosis was associated with a positive PSQ (%) and psychosocial questionnaires (social results, school results, and ADHD index) at follow-up but not with physical abilities.

## 4. Discussion

The current study demonstrated that ODI may be a more accurate predictor than AHI of the long-term neuropsychological and psychosocial consequences of SDB in children. At follow-up, children diagnosed with an ODI of more than one event per hour and persistent respiratory problems during sleep exhibited symptoms in four domains (physical, school-related, quality of life, and ADHD), whereas those with an oAHI of more than one event per hour at diagnosis showed symptoms in only two domains (physical and school-related). These results suggest that ODI (events per hour) may be a better predictor of long-term symptoms than oAHI. Similar findings were observed in children who underwent surgical intervention to treat SDB. Additionally, we found a statistical correlation between the minimum SpO_2_ (%) recorded at diagnosis and various outcomes, including PSQ scores, physical, social, and school-related outcomes, as well as the ADHD index at follow-up. Furthermore, children who underwent surgery and were diagnosed with SDB (ODI > 1 event/h.) and a pathological PSQ at follow-up showed neurobehavioral and neurocognitive impairment in the four domains of physical, educational, QoL, and ADHD, compared to children whose SDB resolved at follow-up. Lastly, oAHI identified symptoms in two domains (physical and QoL).

OSAS is a condition in which airflow is repeatedly blocked during sleep, causing a reduction in the mean oxygen saturation in the blood (SpO_2_). Cerebral blood flow is highly regulated by blood gases (pH, PaO_2_, and PaCO_2_), the autonomic nervous system, and neurovascular coupling. The metabolic regulation of cerebral blood flow should protect the brain from hypoxia, but in patients with SDB, the reactivity of cerebral vessels in response to hypercapnia or hypoxia during sleep has been shown to be compromised [36]. Studies on children with SDB have reported a reduction in gray matter in various regions of the brain [37]. These reductions are likely due to oxidative damage secondary to intermittent desaturations [24]. Free radicals can be more fully explained by ODI than by AHI alone. The impact of intermittent desaturation during sleep on cognitive function in children is more severe because it can affect plastic brain structures, changing neuro-psychic development, learning abilities, and social interactions [25].

Adults with a history of severe childhood OSA have a high risk of snoring, high body mass index, and lower academic performance later in life [19]. In particular, SDB is associated with many comorbidities, including neurobehavioral and neurocognitive impairments [38]. Additionally, the comorbidity between OSAS and ADHD is well recognized [39]. While there are not many studies on the long-term effects of OSAS, the tuCASA survey [5] identified a clear association between OSAS and behavioral disorders, impulse control disorders, attention, and oppositional defiant disorder [7,40]. The Tuscan study also found a negative correlation between AHI and intelligence quotient (IQ) scores, particularly in performance IQ and math scores, while nighttime hypoxemia negatively impacted nonverbal abilities [41]. School problems have been reported in several children with OSA [1], and studies support the relationship between SDB and hyperactive and inattentive behavior [42]. However, not all studies have correlated OSAS severity (AHI) with neurological comorbidity [9].

Despite the abundant research in this area, many questions remain unresolved about the relationship between SDB and attention deficit/hyperactivity, particularly between OSAS and ADHD-like symptoms [1]. Functional alterations in the prefrontal cortex may explain why OSAS affects aspects such as executive functions, memory, motor control, attention, visuospatial skills, learning, and mood regulation [43]. In addition, a study has highlighted the possible role of intermittent hypoxia in children with severe SDB, which can affect aspects beyond physical and emotional intelligence [44].

Both the oAHI and the ODI are parameters used to diagnose OSAS in children, with the oAHI considered the primary parameter [14]. However, a study has shown that the ODI can also be a good parameter to predict the presence and severity of OSAS in children [21]. In this study, the severity of ODI was associated with long-term neuropsychological complications, such as physical, school, quality of life, and ADHD problems, in children with persistent SDB regardless of ENT surgery.

Adenotonsillectomy is considered the first-line treatment in children with SDB. Although adenoid and/or tonsil surgery generally improves cardiorespiratory parameters, it is curative with complete resolution in only 27.2% of children [45]. Nonetheless, not all patients are candidates for surgery, and some will continue to have symptoms after the surgery [46]. Moreover, the effect of surgery on the QoL and neurological functions in this population is highly debated. A meta-analysis suggested that ADHD symptoms were related to SDB and improved after adenotonsillectomy [47]. One study reported significant improvements in cognitive performance [4] if the intervention is performed early enough in the disease, while others show that psychosocial impairment remains over time. Moreover, surgery seems to improve some of these outcomes.

In our study, children who underwent surgery, were diagnosed with SDB (ODI > 1 event/h) and had a pathological PSQ score at follow-up showed neurobehavioral and neurocognitive deterioration in the four domains of physical, education, QoL, and ADHD compared to children whose SDB resolved at follow-up. Therefore, the chronicity of SDB after surgery is associated with the maintenance of neurobehavioral disorders. If the disorder resolves after surgery, neurobehavioral deficits can also regress.

The PSQ is a screening tool that can be used in the follow-up of patients [48]. The percentage of positive results on the PSQ correlates with the severity of neurological comorbidity. A positive PSQ suggests that the patient has SDB and neurological impairment. Furthermore, the PedsQL questionnaire has been used in many studies to analyze the QoL of patients with OSA [33,34]. The PedsQL 4.0 questionnaire effectively identifies the psychosocial involvement of children in follow-up for SDB when evaluating gait is not possible [31]. In addition, QoL was found to be worse in patients with persistent SDB after surgery compared to healthy children, with the most affected functions being physical, and academic. Pediatric SDB has been associated with an increased risk of daytime sleepiness and problematic behaviors indicative of ADHD [48]. However, the behavioral scores were improved statistically significantly even after surgical intervention [49].

In general, our study demonstrates the importance of performing a children check-up to identify cases where the intervention was not effective or where there has been a recurrence of the disorder. Therefore, the PSQ and PedsQL questionnaires can be useful tools for follow-up of children after a surgical intervention, especially during times of restrictions caused by the COVID-19 pandemic, which have led to a reduction in instrumental assessments and an increase in alternative methods such as questionnaires and structured interviews [50].

### Strengths and Limitations

Among the strengths, the ease of performing long-term clinical follow-up of patients is highlighted, verifying the evolution of their health status over time. In addition, the use of multiple tools such as validated questionnaires has ensured the accuracy and validity of the data collected with different standardized questions to investigate various aspects of patients’ health status.

However, the study also has some limitations. Not all parents of the patients adhered to the monitoring and treatment program for their children’s SDB. This may have influenced the study’s results. Additionally, the lack of follow-up PSG data represent another limitation of the study. As PSG is an examination used to diagnose SDB and other sleep disorders, the lack of follow-up data means that we could not accurately verify whether the therapies administered to the patients influenced SDB. Finally, the small sample size of the patients studied may have affected the precision and generalizability of the results obtained.

## 5. Conclusions

Children with an ODI greater than 1 event/h. or a low minimum SpO_2_ (%) at diagnosis are more likely to exhibit long-term physical, school-related, and quality-of-life-related ADHD-type symptoms. Additionally, children who underwent ENT surgery due to pathological ODI and who have pathological PSQ at follow-up will have greater neurobehavioral and neurocognitive signs compared to those with a normal PSQ after surgery. In summary, ODI, minimum SpO_2_ percentage at diagnosis, and PSQ at follow-up can be used as indicators to predict long-term SDB symptoms in children and to monitor treatment effectiveness over time.

## Figures and Tables

**Table 1:** Descriptive statistics for a sample of patients who were contacted, excluded, or included in the study.

	Mean (SD)	Minimum	Maximum	Mean (SD)	Minimum	Maximum	*p*-Value (Mann–Whitney Test)
	Patients Contacted			Patients Included (*)			Patients Included Versus Excluded
N. (males %)	83 (59)			47 (66)	-	-	0.205
Age at PSG (years)	7.0 (3.5)	2.1	14.5	5.8 (2.8)	2.0	13.8	0.022
Weight (kg)	27.0 (21.7)	4.1	125.0	24.4 (15.4)	6.5	102.0	0.200
Height (cm)	112.0 (36.4)	13.1	173.0	113.0 (19.8)	62.0	173,0	0.047
BMI (kg/m^2^)	19.2 (7.2)	12.5	56.0	17.6 (4.4)	12.5	34.1	0.952
BMI z-score	0.3 (2.1)	−9.0	4.1	0.3 (1.7)	−3.8	2.9	0.830
BMI percentile	61.9 (36.4)	0.1	99.9	59.4 (36.2)	0.1	99.8	0.857
**Cardiorespiratory PSG** **(T_0_)**							
Duration of registration (h)	9.2 (1.1)	6.1	13.2	9.3 (0.9)	7.2	11	0.823
OA (events/h)	3.4 (5.5)	0.0	29.3	3.2 (4.6)	0	22	0.705
oAHI (events/h)	5.9 (7.6)	0.0	37.2	4.9 (5.7)	0.0	24.6	0.578
ODI (events/h)	5.4 (7.3)	0.0	36.0	3.2 (4.1)	0.1	23.4	0.354
SpO_2_ mean (%)	96.0 (9.8)	8.0	99.0	97.0 (1.0)	95.0	99.0	0.628
SpO_2_ minimum (%)	85.8 (10.0)	36.0	96.0	88.0 (7.0)	51.0	95.0	0.263
SpO_2_ < 90% (% eTST)	0.7 (1.8)	0.0	13.8	0.3 (0.8)	0.0	4.2	0.600
Snoring (% eTST)	1.6 (3.9)	0.0	22.1	1.5 (4.6)	0.0	22.1	0.986

Legend: BMI, body mass index; eTST, estimated total sleep time; h., hours; OA, obstructive apnea; oAHI, obstructive apnea–hypopnea index; ODI, oxygen de-saturation index; PSG, polysomnography; SD, standard deviation. (*) Adenoidectomy (*n* = 13); tonsillectomy (*n* = 1); adenotonsillectomy (*n* = 13).

**Table 2:** Results of questionnaires administrated to children with oAHI > 1 event/h and in those with ODI > 1 event/h.

T_0_	oAHI > 1 event/h			ODI > 1 event/h		
T_1_	PSQ − (*n* = 12)	PSQ + (*n* = 22)		PSQ − (*n* = 11)	PSQ + (*n* = 21)	
	Mean (SD)	Mean (SD)	*p*-Value (Mann-Whitney Test)	Mean (SD)	Mean (SD)	*p*-Value (Mann–Whitney Test)
Physical results %	92.7 (10.7)	82.4 (13.9)	0.015	92.9 (11.0)	83.2 (14.4)	0.034
Emotional results %	67.9 (15.3)	61.9 (14.7)	0.245	69.5 (14.2)	62.9 (16.8)	0.238
Social results %	82.1 (17.5)	79.1 (21.0)	0.292	85.5 (16.0)	78.1 (20.9)	0.481
Academic achievement %	90.9 (8.8)	74.8 (21.1)	0.048	90.5 (8.8)	71.2 (20.5)	0.009
Psychosocial outcomes %	80.0 (11.1)	71.9 (16.2)	0.169	81.8 (9.3)	70.7 (16.1)	0.056
QoL total %	84.5 (9.9)	75.6 (14.0)	0.063	85.7 (9.1)	75.1 (14.3)	0.038
Objectivity (T-points)	59.3 (13.5)	67.3 (16.0)	0.245	60.5 (14.0)	64.9 (16.1)	0.639
Cognitive disorders (T-points)	55.3 (14.4)	59.7 (15.6)	0.511	54.6 (15.2)	62.3 (15.1)	0.144
Hyperactivity (T-points)	58.5 (15.2)	64.1 (12.8)	0.217	57.2 (16.3)	65.7 (12.6)	0.113
ADHD index (T-points)	61.8 (13.0)	68.0 (12.6)	0.179	58.7 (11.8)	70.7 (12.4)	0.011

Legend: ADHD, attention deficit hyperactivity disorder; oAHI, obstructive apnea–hypopnea index; ODI, oxygen desaturation index; PSQ, Pediatric Sleep Questionnaire; QoL, Quality of Life; SD, standard deviation; T_0_, time of which children performed respiratory polygraphy; T_1_, time of which children fulfilled the questionnaire (follow-up).

**Table 3:** Follow-up of children who and underwent a surgical intervention (adenoidectomy n.11; tonsillectomy n.1; adenotonsillectomy n.10) with ODI > 1 event/h or oAHI > 1 event/h.

T_1_	oAHI > 1 event/h			ODI > 1 event/h		
T_0_	PSQ − (*n* = 10)	PSQ + (*n* = 12)		PSQ − (*n* = 8)	PSQ + (*n* = 12)	
	Mean (SD)	Mean (SD)	*p*-Value (Mann–Whitney test)	Mean (SD)	Mean (SD)	*p*-Value (Mann-Whitney Test)
Physical results (%)	97.2 (4.3)	82.6 (16.0)	0.007	98.4 (2.9)	83.1 (16.5)	0.012
Emotional results (%)	69.5 (15.7)	60.5 (15.5)	0.180	74.4 (13.4)	63.8 (19.3)	0.157
Social outcomes (%)	86.5 (14.9)	80.0 (21.2)	0.771	91.3 (10.6)	80.0 (21.2)	0.473
Educational attainment (%)	90.0 (8.5)	74.2 (21.2)	0.123	91.3 (8.8)	72.5 (19.7)	0.039
Psychosocial outcomes (%)	82.0 (10.1)	71.6 (14.8)	0.123	85.6 (5.6)	72.1 (15.6)	0.057
QoL total score (%)	87.3 (7.9)	75.4 (14.3)	0.030	90.1 (4.3)	75.9 (14.9)	0.020
Objectivity (T-score)	57.2 (9.7)	70.5 (19.6)	0.203	56.8 (9.8)	67.3 (19.7)	0.473
Cognitive disorders (T-score)	51.0 (7.5)	61.5 (17.7)	0.203	50.4 (8.0)	62.2 (17.2)	0.135
Hyperactivity (T-score)	57.1 (13.9)	66.1 (13.7)	0.159	56.4 (15.7)	66.5 (13.7)	0.157
ADHD index (T-score)	59.5 (10.8)	69.2 (15.1)	0.159	55.9 (7.1)	71.5 (14.7)	0.020

Legend: ADHD, attention deficit hyperactivity disorder; oAHI, obstructive apnea–hypopnea index; ODI, oxygen desaturation index; QoL, quality of life; PSQ, pediatric sleep questionnaire.

**Table 4:** Multiple regression analysis exploring the relationship between SDB indices and questionnaire domains. Beta indicates the effect of each independent variable on the value of the dependent variable, while holding the other variables constant. The t-value and *p*-value indicate whether each independent variable has a significant effect on the value of the dependent variable. Confidence intervals (95% C.I. for B) indicate the range of values within which the regression coefficient is expected to occur with a certain probability.

Dependent Variable	Variables Included in the Model	T	S.E.	Beta	t	Significance (*p*)	C.I. for B 95% (Lower Limit)	C.I. for B 95% (Upper Limit)
PSQ score (%), T_0_	Physical results (%)	−0.543	0.226	−0.360	−2.406	0.021	−0.998	−0.087
	Emotional results (%)	−0.353	0.153	−0.288	−0.304	0.026	−0.662	−0.044
	Social results (%)	0.316	0.169	0.295	1.864	0.069	−0.026	0.658
	School results (%)	−0.435	0.139	−0.429	−3.125	0.003	−0.716	−0.154
	Opposition (T-points)	0.323	0.14	0.238	2.230	0.031	0.030	0.626
oAHI (events/h), T_0_	Physical results (%)	−0.092	0.064	−0.209	−1.432	0.015	−0.221	0.037
ODI (events/h), T_0_	Physical results (%)	0.029	0.047	0.093	0.624	0.536	−0.065	0.123
Tempo SpO_2_ (%), T_0_	Physical results (%)	0.010	0.009	0.163	1.111	0.272	−0.008	0.028
SpO_2_ min (%), T_0_	PSQ (%)	−0.161	0.062	−0.439	−2.605	0.013	−0.286	−0.036
	Physical abilities (%)	−0.257	0.104	−0.464	−2.475	0.018	−0.467	−0.047
	Social abilities (%)	0.190	0.074	0.483	2.552	0.015	0.040	0.340
	School abilities (%)	0.159	0.071	0.429	2.235	0.031	0.015	0.304
	Index ADHD (T-points)	0.259	0.092	0.536	2.810	0.008	0.073	0.445
BMI z-score, T_0_	Social results (%)	−0.013	0.014	−0.142	−0.963	0.341	−0.041	0.015

Legend: BMI, body mass index; oAHI, obstructive apnea–hypopnea index; ODI, oxygen desaturation index; PSQ, pediatric sleep questionnaire.

## Data Availability

Data sharing not applicable.
